# Treatment of Slight Wounds

**Published:** 1891-06

**Authors:** 


### TREATMENT OF SLIGHT WOUNDS.

In the treatment of wounds we must attend to these rules : First, stanch the bleeding ; secondly, put the injured part under the most favorable conditions for healing to occur ; and, lastly, we must try to prevent excessive inflammatory action from setting in, and avoid blood poisoning.

If the cut is a small one, a very little pressure will stop the bleeding, such as that of a piece of rag tied moderately tight round the part. If larger, a piece of soft rag, or lint, must be folded up, made into a pad, pressed on the bleeding part, and tied there.

In large wounds, and where blood is coming out in small jets with every beat of the heart, the fingers must be firmly pressed on the part for some time, until a clot forms in the vessel and blocks it up. If a very large blood-vessel has been cut and there is danger of the person bleeding to death, the usual flow of blood to the part must be nearly stopped. This is readily done by firmly tying a hankerchief or scarf round the limb between the wound and the heart. If this does not arrest the bleeding, a piece of stick must be put under the tight bandage, and turned round and round until the handkerchief or scarf is made so tight as to stop the loss of blood. The patient must then be quietly conveyed to the nearest surgeon or hospital with this tight appliance on the limb. After stanching the bleeding, the next condition is to secure the most favorable state for healing. This is done by bringing the cut tissues together, and keeping them at rest.

In small wounds a bit of rag will keep the pieces of cut skin close together, but in larger wounds the edges must be mechanically brought together. Some do this by pieces of sticking-plaster, but more satisfactory results are got by putting in a few stitches. Any one with a little nerve can put stitches into the edges of a wound, bring them together, and keep them in contact by tying the thread. An ordinary needle will do; thread it with silk, push through the skin from the outside of the wound and about one-twelfth of an inch from its edge, draw the thread partly through, then pierce the skin of the opposite side from the inside of the wound, bring the two edges together, tie the thread with two or three knots, and the operation is complete. Put the stitches about a quarter of an inch apart, and sew up all the wound. Use a clean needle, uncolored silk thread, and dip both into boiling water before using.

